# The fibrotic and immune microenvironments as targetable drivers of metastasis

**DOI:** 10.1038/s41416-020-01172-1

**Published:** 2020-11-26

**Authors:** Luke Boulter, Esme Bullock, Zeanap Mabruk, Valerie G. Brunton

**Affiliations:** 1 MRC Human Genetics Unit, Institute of Genetics and Molecular Medicine, University of Edinburgh, Crewe Road South, Edinburgh, EH4 2XU UK; 2 Edinburgh Cancer Research UK Centre, Institute of Genetics and Molecular Medicine, University of Edinburgh, Crewe Road South, Edinburgh, EH4 2XU UK

**Keywords:** Cancer microenvironment, Mechanisms of disease

## Abstract

Although substantial progress has been made over the past 40 years in treating patients with cancer, effective therapies for those who are diagnosed with advanced metastatic disease are still few and far between. Cancer cells do not exist in isolation: rather, they exist within a complex microenvironment composed of stromal cells and extracellular matrix. Within this tumour microenvironment exists an interplay between the two main stromal cell subtypes, cancer-associated fibroblasts (CAFs) and immune cells, that are important in controlling metastasis. A complex network of paracrine signalling pathways between CAFs, immune cells and tumour cells are involved at multiple stages of the metastatic process, from invasion and intravasation at the primary tumour site to extravasation and colonisation in the metastatic site. Heterogeneity and plasticity within stromal cell populations also contribute to the complexity. Although many of these processes are likely to be common to a number of metastatic sites, we will describe in detail the interplay within the liver, a preferred site of metastasis for many tumours. A greater understanding of these networks provides opportunities for the design of new therapeutic approaches for targeting the metastatic disease.

## Background

Although significant advances have been made over the past 40 years in the treatment of cancer, most patients with advanced metastatic disease are faced with the harsh reality that no effective treatments currently exist. As such, the majority of cancer-related deaths are associated with metastatic spread.^1,2^ The key to advancing new treatment options is a greater understanding of the complex network of biological processes that control the metastatic process.

**Fig. 1:**
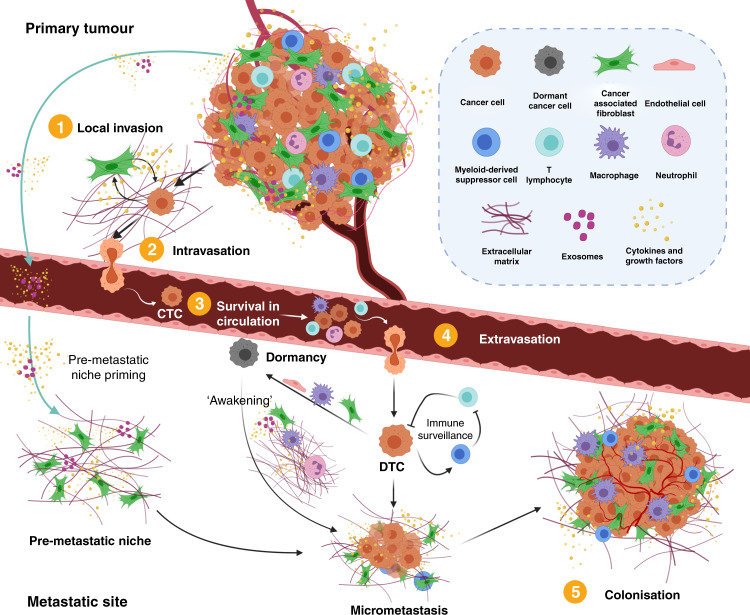
The metastatic cascade. Metastasis occurs in a number of steps starting with the invasion of tumour cells into the local stroma (1) and their intravasation (2) into the vascular or lymphatic system. In the case of haematogenous metastasis, they must survive the mechanical forces of the circulatory system and suppressive immune cells (3). Few circulating tumour cells (CTCs) survive the circulation but those that do then extravasate into the metastatic site (4). The systemic release of exosomes, inflammatory cytokines and growth factors by cancer and stromal cells in the primary tumour can ‘prime’ and remodel the microenvironment of distant organs to form a pre-metastatic niche that supports the outgrowth of DTCs before their arrival.^14^ Once in the metastatic site, disseminated tumour cells (DTCs) must overcome immune surveillance by resident immune cells, and can direct immune suppression by recruiting myeloid-derived suppressor cells. DTCs can also become dormant, induced by factors derived from metastatic niche stromal cells,^110–112^ or supported by signalling from endothelial cells,^113^ and then subsequently ‘awakened’ by signals from surrounding stromal cells and the extracellular matrix (ECM).^15,114^ Reawakening of dormant DTCs can also be induced by exosomes, either released by metastatic niche stromal cells or systemically released by cancer-associated fibroblasts (CAFs) in the primary site.^38,115^ Proliferating DTCs form micrometastases and, finally, colonisation of the metastatic niche occurs and tumour cells form clinically relevant macrometastases, co-opting and recruiting local stromal cells to support metastatic cell growth (5). Created with BioRender.com.

Metastasis occurs in several stages, and involves the accumulation of genetic, epigenetic and metabolic alterations in tumour cells, alongside complementary changes in, and signalling from, the tumour microenvironment (TME).^2,3^ To initiate the first step of the metastatic cascade—invasion into the local stroma—tumour cells must become motile and invasive, which requires changes in cell–cell and cell–extracellular matrix (ECM) contacts as well as reorganisation of the ECM. Tumour cells can then intravasate, breaking through the basement membrane of the vasculature or into lymphatic vessels. Once in the circulatory or lymphatic system, the newly defined circulating tumour cells (CTCs) must survive exposure to mechanical force and the immune system. CTCs can become trapped in small circulatory beds in the lungs and liver, for example—common metastatic sites for multiple cancer types—where they can adhere to endothelial cells and extravasate into the surrounding tissue. Tumour cells in metastatic sites are known as disseminated tumour cells (DTCs) and can either proliferate to form micrometastases or enter a dormant state, from which they can be ‘awakened’ after long periods of latency. The final step in the metastatic cascade, colonisation, occurs when DTCs proliferate to form clinically relevant metastases (Fig. 1). Whereas tumours used to be thought of as a clonal population of one malignant cell type, we now know that they behave more like a tissue, with heterogeneous malignant cells co-operating with multiple different stromal cell types and ECM components in the surrounding TME. Cellular components of the TME include cancer-associated fibroblasts (CAFs), endothelial cells, adaptive and innate immune cells, and non-transformed epithelial cells, with ECM proteins providing physical support and orchestrating intercellular cues. The TME contributes to all the recognised hallmarks of cancer: sustaining proliferative signalling, evading growth suppressors, resisting cell death, enabling replicative immortality, inducing angiogenesis, and activating invasion and metastasis.^4^ When Welch and Hurst^3^ defined the hallmarks of metastasis, they identified ‘the ability to modulate the secondary site or local microenvironments’ as a characteristic of all metastases. Multiple bi-directional signalling axes between tumour cells and the TME drive the multifaceted metastatic process. A complex network of paracrine signalling pathways between different stromal cell types, including CAFs, immune cells, and endothelial and myeloid cells, which can drive metastases, has been identified in both the primary tumour and metastatic sites.^5–9^ In addition, stromal cells such as CAFs and macrophages secrete a number of factors including extracellular proteases and protease inhibitors that are involved in remodelling the ECM.^6,10,11^ Although the composition of the ECM varies dramatically between cancer types, a common remodelling programme of the ECM and ECM-associated proteins (known as the matrisome) has been suggested to occur during metastasis in multiple solid carcinomas, and the pattern of expression of 22 so-called ‘matrisome’ genes was shown to be predictive of disease outcome.^12,13^ The primary tumour can also direct the formation of a ‘pre-metastatic niche’ via the systemic release of tumour-derived factors and extracellular vesicles that result in the restructuring and ‘priming’ of the metastatic site prior to the arrival of tumour cells (reviewed in ref. ^14^). The TME within these metastatic niches provides survival cues for the tumour cells, including immune-suppressive signals that determine whether DTCs form metastases or remain dormant, and can also direct the reactivation of dormant cells to mediate late recurrences in patients (Fig. 1).^6,15^

Understanding the relationship between the various elements of the TME is key to fully elucidating the biological processes that drive the metastatic cascade, which, in turn, should provide opportunities for therapeutic intervention. In this review, we will introduce the interplay between different cellular components of the TME, with a focus on CAFs and immune cells, and describe significant advances made over the past 5 years detailing the role of these components in mediating metastatic spread. Although many of these processes are likely to be common to a number of metastatic sites, we will describe in detail the metastatic microenvironment in the liver, a preferred site of metastasis for many tumours, including colorectal, pancreatic tumours, melanomas and sarcomas.^16^

## Key cellular players in the TME: CAFs

### The origin of CAFs

Due to the lack of cell markers that are unique to fibroblasts and CAFs, the accurate definition of fibroblasts and CAFs remains elusive, as does the origin of CAFs. Classically, fibroblasts are defined as spindle-shaped cells that are embedded within the ECM and lack epithelial, endothelial and leucocyte markers. In the normal physiological stroma, fibroblasts exist in a quiescent state; however, in the reactive tumour stroma, multiple signals can push resting fibroblasts to an activated state. These signals include inflammatory cytokines and growth factors such as transforming growth factor-β (TGF-β) and platelet-derived growth factor (PDGF) secreted from tumour cells and other stromal cells within the TME. In addition, mechanical changes and remodelling of the ECM can drive CAF activation. Although, in most cases, CAFs originate from pre-existing quiescent fibroblasts, in some tissues, activated CAF-like populations can also derive from additional tissue-resident precursors, such as hepatic stellate cells (HSCs; as detailed below), bone marrow-derived mesenchymal stem cells, adipocytes and pericytes.^10,11^

### Pro-tumorigenic and pro-metastatic roles of CAFs

**Fig. 2:**
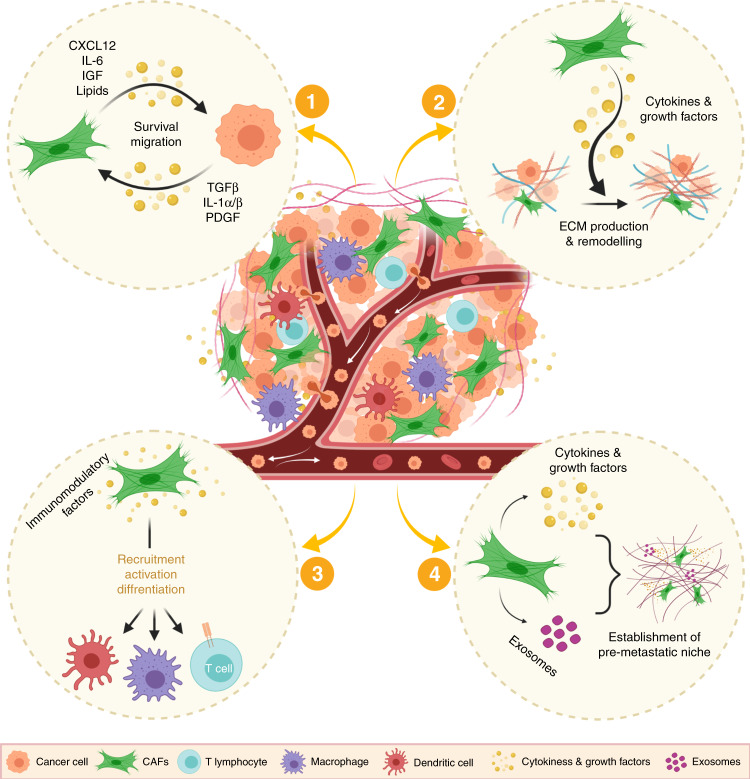
Metastasis-promoting functions of cancer-associated fibroblasts. Heterogeneous cancer-associated fibroblasts (CAFs) drive pro-tumorigenic and metastatic phenotypes via diverse mechanisms: (1) secretion of soluble factors such as growth factors, cytokines and lipids to promote tumour survival and metastasis; (2) secretion and remodelling of components of the extracellular matrix (ECM); (3) secretion of immunomodulatory factors, which contribute to changes in the tumour immune microenvironment; and (4) establishment of a pre-metastatic niche through the secretion of soluble factors and extracellular vesicles. Created with BioRender.com.

A plethora of studies have demonstrated that CAFs are critical regulators of tumour growth and metastasis, acting via multiple mechanisms (Fig. 2). They can secrete soluble factors such as growth factors, cytokines and lipids to promote growth and survival through paracrine signalling in the tumour environment, as well as secreting and remodelling ECM components. The secretion of factors at the primary site, along with matrix remodelling and changes in mechanotransduction, can promote epithelial-to-mesenchymal transition (EMT), migration and invasiveness,^17,18^ while the release of circulating factors such as TGF-β can also promote metastatic outgrowth.^19^ Importantly, CAFs have a role in shaping the immune environment at primary and metastatic sites. Moreover, CAFs can facilitate the systemic delivery of soluble factors and extracellular vesicles to prime the metastatic niche in an organ-specific manner.^14^ Within the metastatic niche, CAFs can also directly influence DTCs to promote their growth and colonisation. Unsurprisingly, therefore, a number of studies have attributed pro-metastatic functions to CAFs in various tumour models.^18–23^

Despite the well-established pro-tumorigenic activity of CAFs, a tumour-suppressive CAF phenotype has also been reported in mouse models of pancreatic ductal adenocarcinoma (PDAC).^24,25^ An increased understanding of heterogeneity within the stromal compartment (below) might help us to shed light on the multifaceted roles of CAFs in balancing pro- and anti-tumour effects.

### CAF functional and spatial heterogeneity

Several studies have revealed the existence of distinct CAF subtypes—as defined by their expression of surface markers—that display functional heterogeneity and might also be tumour type-dependent.^26–28^ These markers include fibroblast activation protein (FAP), fibroblast-specific protein 1 (also known as S100A4) and PDGF receptor-β. However, the characterisation of CAFs is further complicated by reports that these markers can also be expressed in tumour cells that are undergoing EMT.^29^

Distinct CAF subtypes also affect tumour invasion and metastasis. For example, a subset of CAFs is able to drive tumour progression through integrin α11-mediated promotion of CAF invasion and CAF-induced tumour cell invasion via the production of the pro-invasive matrix protein tenascin C.^30^ Furthermore, in breast cancer, a CD146^+^-fibroblast population could preferentially increase metastasis associated with the deposition of a number of ECM components found in clinically aggressive disease.^31^ An analysis of lymph node metastases from breast cancer patients identified divergent CAF subsets that were similar to those found in primary tumours. These subsets were shown to promote metastases through complementary mechanisms: either through their highly contractile phenotype and activation of NOTCH signalling or through the induction of EMT resulting from C-X-C motif chemokine ligand 12 (CXCL12) and TGF-β signalling.^32^ Education of subsets of CAFs by tumour cells to drive a pro-metastatic niche suggests that, in addition to the spatial context of CAFs with respect to tumour cell proximity,^33^ the tumour genotype plays an important role in the regulation of CAF heterogeneity.^27^ In this context, the p53 status in pancreatic tumours can reprogramme a subset of CAFs to establish a metastatic-permissive environment.^34^

Several reports have identified CAF populations within metastatic sites that exhibit distinct gene expression patterns compared with those in the primary tumour.^35,36^ This additional level of CAF spatial heterogeneity has been associated with unique metastasis-enhancing phenotypes. For example, CAFs generated from breast cancer brain metastasis were able to induce the migration of patient-derived tumour cells through the expression of CXCL16 and CXCL12.^35^ In addition, the selective upregulation of the insulin-like growth factor 2 gene *IGF2* and interferon-related genes in CAFs from different breast cancer metastatic tissues compared with primary-site derived CAFs facilitated CAF-dependent tumour formation and metastasis in vivo, and immunosuppression via regulation of T cells.^36^

### Metabolic support from CAFs

Other studies have also highlighted the importance of CAFs in providing metabolic support to drive metastasis. In an ovarian cancer co-culture model, bi-directional signalling between CAFs and tumour cells leads to the mobilisation of glycogen as an energy source in cancer cells, which facilitates metastatic outgrowth.^37^ Furthermore, the horizontal transfer of entire mitochondrial genomes from CAF-derived extracellular vesicles to breast cancer cells treated with hormone therapy restores oxidative phosphorylation, promoting metastasis and reawakening therapy-induced dormant cells.^38^ The exchange and hydrolysis of intracellular lipids provide another approach by which CAFs support tumour progression. PDAC-derived CAFs secrete abundant amounts of lysophosphatidylcholines (LPCs), which can be taken up by PDAC cells, leading in turn to the production of lysophosphatidic acid (LPA), a growth-permissive phospholipid, via the activity of autotaxin. This stroma-derived LPC–autotaxin–LPA axis was found to fuel PDAC growth and migration, although any effects on metastatic progression have yet to be established.^39^ Metabolic differences among patient-derived melanoma xenograft models driven by differences in the levels of monocarboxylate transporter 1, a lactate transporter, are associated with differing metastatic potential,^40^ highlighting the importance of metabolic alterations in regulating metastatic progression and the potential for new therapeutic opportunities. It will be important to consider how dysregulated metabolic signalling in the CAFs might also influence such treatments.

## Key cellular players in the TME: immune cells

The immune system can influence all stages of the metastatic cascade, from the regulation of tumour cell migration and invasion at the primary tumour site through to priming of the metastatic niche and supporting the outgrowth of DTCs (Fig. 1).^8,9^

### Immune cells and the metastatic cascade

In primary tumours, immune cells are important regulators of the ECM and secrete a number of pro-tumorigenic and pro-metastatic proteases, such as metalloproteinases, that remodel the ECM.^41,42^ Immune cells also secrete factors that promote tumour cell intravasation at the primary tumour site.^43^ Further along in the metastatic process, the recruitment of inflammatory monocytes at metastatic sites promotes vascular permeability and extravasation of CTCs.^44^ Moreover, metastasis-associated macrophages derived from these inflammatory monocytes can further enhance metastatic outgrowth through the secretion of cytokines, and several studies have identified specific subsets of macrophages that are involved in organ-specific metastatic outgrowth.^45,46^ Neutrophil-derived factors also promote the extravasation of CTCs, and the release of neutrophil traps further acts to enhance the trapping of CTCs at distant metastatic sites, leading to increased metastatic spread.^47^ Neutrophils also play an important role in promoting metastatic colonisation: they help to create an immunosuppressive environment by secreting nitric oxide, which inhibits T cell functions.^48^ However, anti-metastatic effects of neutrophils have also been described. Work published by Li et al.^49^ in 2020 attempted to address these differences and highlighted the importance of natural killer (NK) cells in dictating whether neutrophils exert pro- or anti-metastatic effects. In NK cell-competent mice, neutrophils facilitate metastatic colonisation, while in NK cell-deficient mice, neutrophils have an inhibitory effect on metastatic colonisation.

Immune cells also play a pivotal role in establishing a permissive pre-metastatic niche by secreting a number of chemoattractants, while tumour-derived factors stimulate the mobilisation and recruitment of bone marrow-derived myeloid cells to the metastatic niche to remodel the ECM, promote angiogenesis and drive a pro-inflammatory environment—all of which contribute to metastatic seeding and colonisation.^50^

### The influence of CAFs on the inflammatory environment

CAFs also play a key role in regulating the inflammatory tumour environment through the secretion of cytokines and deposition of ECM proteins that are involved in the recruitment and activation of immune cells.^51^ Important bi-directional crosstalk between tumour cells and CAFs controls the balance between the recruitment of pro-tumorigenic and anti-tumorigenic immune cell populations, which influences both primary tumour growth and metastatic progression. A number of studies have provided further insight into the complex signalling pathways involved. For example, CAFs act as sensors of damage-associated molecular patterns produced within primary tumours, which activates the NLRP3 inflammasome pathway and induces the subsequent secretion from CAFs of the pro-inflammatory cytokine interleukin-1β (IL-1β). IL-1β, in turn, promotes metastases through multiple mechanisms, among which is a reduction in the recruitment of immunosuppressive CD11b^+^Gr1^+^ myeloid cells in the metastatic environment.^52^ Conversely, DTCs can induce a pro-inflammatory phenotype in fibroblasts within the metastatic niche by secreting IL-1α and IL-1β, which trigger the production by fibroblasts of CXCL9 and CXCL10 to subsequently support the outgrowth of the DTCs.^53^

## Key non-cellular components in the TME: the ECM and its remodelling

The non-cellular component of the TME, the ECM, is a network of macromolecules, including collagens, fibronectin and elastin, which provides structural support in a tissue-specific manner. It is a highly dynamic structure that responds to external cues, while also providing biochemical and biomechanical signals to surrounding cells within the tissue, thereby playing a fundamental role in normal tissue homeostasis and disease processes including metastasis formation.^54^ Detailed review of the role of the ECM in metastasis is beyond the scope of this article and has been reviewed elsewhere.^13^ Here, we highlight the role of CAFs and immune cells in regulating ECM remodelling.

The concept of dynamic reciprocity, whereby cells process signals from their environment, which can then, in turn, regulate the ECM, was first introduced in the early 1980s in the context of epithelial morphogenesis and wound healing, and has since been identified as a crucial process regulating the behaviour of tumours, especially the invasive and metastatic potential of tumour cells.^55^ This reciprocity is predominantly mediated via cell-surface ECM receptors, which include integrins and collagen-binding discoidin domain receptors (DDRs), and both CAFs and macrophages play a key role in this process leading to remodelling of the ECM in the metastatic niche to promote cell adhesion and survival.

A number of new players in the CAF-dependent remodelling of the ECM that control mechanotransduction and ECM stiffness to drive metastasis have been identified. The deletion in CAFs of DDR2, a critical regulator of integrin-based mechanotransduction and collagen fibre organisation, results in a significant reduction in tumour stiffness and the extent of pulmonary metastasis.^56^ In a model of melanoma, a novel role for fibroblast-secreted factors in altering the lymphatic ECM has been identified. Secretion of hyaluronic and proteoglycan link protein (HAPLN1), which crosslinks hyaluronan to the ECM, leads to changes in the permeability of lymphatic endothelial cells, thereby enabling melanoma cells to escape from the lymphatic system to distant metastatic sites.^57^ Bertero et al.^58^ have also shown that changes in ECM stiffness induce changes in amino acid metabolism in both CAFs and tumour cells, and that these changes drive a complex crosstalk whereby CAF-derived aspartate sustains cancer cell proliferation, while cancer cell-derived glutamate balances the redox state of CAFs to promote ECM remodelling. Targeting aspartate and glutamate transporters either genetically or pharmacologically reduced metastatic spread.

The interaction between CAFs, immune cells and remodelling of the ECM is of particular importance in the fibrotic liver environment, and we will now discuss in detail the interplay between metastatic tumour cells, the immune environment, and HSCs (the resident fibroblasts within the liver) in the liver environment, a common site of metastasis.

## The formation of a metastatic microenvironment in the liver

Following metastatic spread, cancer cells from a number of primary malignancies find themselves in the liver, which appears to be a preferred site of metastasis for many tumours.^59–61^ Why the liver is such a privileged site is a topic of much debate. Nevertheless, for a metastatic cell to take hold, the recipient organ requires an extensive vascular network to provide ready access for the cancer cell. Moreover, to establish a secondary tumour, the cancer cells must be able to take over a dynamic local immune environment that it can utilise to facilitate its growth.

### Sinusoids: cellular suppressors of metastasis

**Fig. 3:**
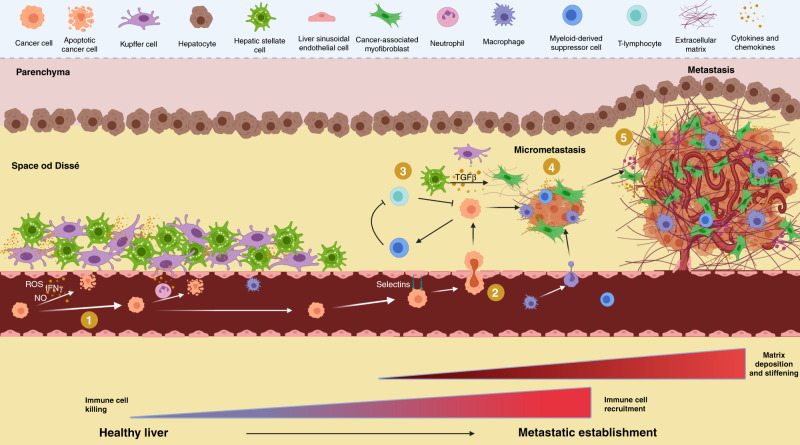
The metastatic microenvironment in the liver. The liver provides a permissive environment for metastatic colonisation. (1) Circulating tumour cells (CTCs) within the liver microvasculature must survive immune attack from circulating immune cell populations. (2) Cells then enter the lumen of the hepatic sinusoid via specialised sieve-like fenestrated endothelial cells (liver sinusoidal endothelial cells; LSECs). (3) Cells are subjected to further immune surveillance mediated via cytokine and chemokine crosstalk between sinusoidal endothelial cells, Kupffer cells and lymphocytes. (4) The tumour cells must then establish themselves within the space of Dissé, where they recruit hepatic stellate cells (HSCs), myeloid-derived suppressor cells and lymphocytes to establish a metastatic-promoting environment required for the initiation of micrometastases. (5) The further outgrowth of metastases is accompanied by matrix deposition and stiffening mediated by activated HSCs and the formation of a local vasculature and recruitment of pro-inflammatory immune cells. Created with BioRender.com.

The vasculature of the liver is highly specialised, with blood entering through the portal circulation and passing through sinusoids. These narrow vessels, which are lined with sieve-like fenestrated endothelial cells (liver sinusoidal endothelial cells; LSECs), form a highly permeable barrier that facilitates the rapid exchange of biological and xenobiotic compounds between the blood and the metabolic hepatocytes. As blood passes through this highly efficient filtration system, it is surveyed by local populations of immune cells adjacent to the LSECs, including Kupffer cells (resident hepatic macrophages) and other circulating lymphocytes (Fig. 3).^62^ Within the lumen of hepatic sinusoids, CTCs have the potential to extravasate to initiate a metastasis; however, relatively few do. Although a number of CTCs might pass through the hepatic sinusoids, many will be killed by a complex sinusoidal immune surveillance network.^63,64^ Importantly, the sinusoids function as active cellular suppressors of metastasis, with cytokine and chemokine crosstalk between sinusoidal endothelial cells, Kupffer cells, and circulating lymphocytes acting together to identify CTCs and mark them for cellular killing.^65^ This multicellular approach involves the release of pro-apoptotic factors, such as reactive oxygen species, nitric oxide and interferon-γ, by sinusoidal endothelial cells, thereby inducing the direct apoptosis of CTCs.^66^ In tandem with this, Kupffer cells lining the sinusoids are able to detect the presence of cancer cells and, through the release of a number of pro-inflammatory cytokines, recruit a transient lymphocyte niche that senses and targets metastatic cells, thereby clearing them from the microvasculature and preventing them from establishing a new metastatic tumour.^67,68^

### Surmounting the sinusoids

The initiation of metastasis in the liver requires cancer cells to cross the immunologically defensive barrier of endothelial cells and immune cells, and establish themselves within the space of Dissé, a region adjacent to the sinusoid which, in healthy individuals, accommodates HSCs—liver fibroblasts that are central for establishing scar formation following injury (Fig. 3). Somewhat counterintuitively, many of the cell types that are required for immune surveillance and the formation of a physical barrier to prevent metastatic colonisation of the liver are co-opted by cancer cells. Several studies have highlighted the importance of cell adhesion molecules such as integrins and selectins on sinusoidal endothelial cells, which immobilise cancer cells as they move through the sinusoids. This strategy enables the CTCs to delaminate through the endothelial layer of the sinusoid and into the space of Dissé, where they are protected from immediate immune sensing and the processes of tumour cell clearance.^69–71^

### Generating a vascular network

Once in the space of Dissé, tumour cells are able to recruit myeloid-derived suppressor cells, which can then modulate the tumour cell immune microenvironment by, for example, suppressing CD8^+^ T cells, thereby promoting colonisation of the liver with cancer cells.^72^ However, the formation of micrometastases in the space of Dissé is limited by the availability of a vascular network. Evidence suggests that, in the liver, these DTCs can promote the formation of a local vasculature from the sinusoidal endothelial cells or from the larger vessels in the liver; whether one of these vascular origins is favoured is not clear.^73,74^ However, the dynamic recruitment of a vasculature necessitates a change in the local immune microenvironment, and again, high levels of TGF-β (produced by the cancer cells) can promote the polarisation of metastasis-associated macrophages to an M2-like phenotype (alternatively activated macrophages) while suppressing M1 (classically activated macrophages) tumour necrosis factor-α (TNF-α)-producing macrophages.^64,75–77^ This immunological switching of immune cell phenotypes is considered to be an essential trait of tumour initiation and, as a consequence, M2-like macrophages drive remodelling of the ECM through the production of matrix metalloproteinases, as well as supporting fibroblasts to produce pro-angiogenic signals such as vascular endothelial growth factor (VEGF) to facilitate further vascular recruitment.^78,79^

### Role of HSCs

In addition to a complex immune microenvironment, the liver also contains two distinct populations of fibrogenic cells: HSCs in the space of Dissé^80^ and portal fibroblasts surrounding the bile duct.^81^ Fibroblasts are normally quiescent but, in response to injury, both of these cell types are capable of activation and express classical markers of activated fibroblasts such as α-smooth muscle actin (α-SMA), tissue inhibitors of metalloproteinases and ECM components. In premalignant, fibrotic disease, HSCs are activated following exposure to a number of extracellular cytokines (reviewed in ref. ^82^) and through a number of cell-autonomous processes.^83^ The plasticity of these cells and their relative contribution to premalignant disease, however, might be more dynamic than previously thought, and only since the application of single-cell RNA-sequencing to these populations has their complexity been appreciated (reviewed in ref. ^84^).

Activated HSCs (also known as hepatic myofibroblasts) contribute to scar formation and fibrosis in chronic liver disease by depositing collagen and other components of the scar.^85^ This stiffening of the liver is thought to make the tissue permissive to cancer cell proliferation and, in primary liver cancer, HSCs are a known source of many mitogens and cytokines that are required for cancer growth.^86,87^ In metastatic disease too, HSCs appear to play a reiterative role in metastatic priming and tumour progression.

The de novo production of highly crosslinked collagen in this new metastatic environment has two principal effects. Initially, it promotes survival by stiffening the local environment, and a number of studies have linked increased stiffness of the ECM to enhanced tumour cell survival in the liver and increased progression through the cell cycle. Shen et al.^88^ reported in 2020 that activated fibroblasts isolated from liver metastases, which express α-SMA, phosphorylated myosin light chain and collagen-1, are highly contractile and increase the local rigidity surrounding the metastasis, thereby supporting angiogenesis and metastatic growth. Furthermore, the altered ECM promotes pro-survival signalling through the engagement of ECM receptors such as integrins,^71^ thereby conferring a survival advantage on tumour cells within the space of Dissé over those that remain within the sinusoid.

The transition of quiescent HSCs into activated fibroblasts also controls PDAC quiescence in the hepatic niche. Whereas cancerous epithelial cells from PDAC are forced into IL-8-induced quiescence by quiescent HSCs, activated HSCs fail to induce cancer cell quiescence, but rather support cancer cell proliferation by expressing growth factors such as VEGF,^78^ and are sufficient to promote a stem-like phenotype in pancreatic cancer cells by downregulating E-cadherin and inducing the expression of mesenchymal markers and nestin. This stem-like phenotype is partially reversed by inhibiting TNF-α signalling between the HSCs and tumour cells.^89^

The quiescent HSC to active HSC transition is clearly important in allowing the liver to be permissive to colonisation by pancreatic cancer cells. Similar mechanisms are likely to play a role in the colonisation of the liver by other tumour cells, too. Importantly, should the process by which these different tumour cells colonise the liver be a common one, then developing methods to intervene in this would be far-reaching. Relaxin, an endogenous peptide hormone, has anti-fibrotic properties and has been shown to be effective in ameliorating scar formation in the liver and reversing systemic syndromes associated with liver stiffening in cirrhosis. Interestingly, in mouse models of liver colonisation by pancreatic, colorectal and breast cancer cells, the production of ectopic relaxin reduces the activation of HSCs at the metastatic site and inhibits metastatic colonisation. Moreover, when the activation of HSCs is reduced, metastases are more susceptible to treatment with immunotherapy,^90^ placing HSCs and activated macrophages at the centre of a microenvironment that can modulate both the growth of metastases and also their immune surveillance.

### Priming the hepatic metastatic niche

As mentioned, the liver appears to be a privileged site for cancer metastasis. As such, there has been a significant focus on how a metastatic microenvironment is established within the liver, whether this microenvironment is essential for the seeding of metastatic cancer within the liver, and whether primary tumours themselves are able to remotely alter the liver metastatic microenvironment to make it receptive to colonisation. In PDAC, tumour-derived exosomes mediate the formation of a pre-metastatic microenvironment in the liver, which then facilitates colonisation by metastatic cancer cells.^91^ The exosomes, which contain high levels of macrophage migration inhibitory factor (MIF), are taken up by Kupffer cells, which are induced to express TGF-β. These TGF-β-expressing macrophages subsequently activate local HSCs to produce fibronectin, thereby generating a local microenvironment that is permissive for the recruitment of bone marrow-derived macrophages that is required for the formation of the pre-metastatic niche and metastatic outgrowth. Inhibition of exosomal-derived MIF leads to reduced colonisation of the liver with metastatic cancer cells, highlighting the importance of distant cues in priming the liver to receive metastatic cells. Subsequent studies have indicated that the population of bone marrow-derived monocytes that is recruited to the site of metastasis comprises pro-inflammatory monocytes that secrete high levels of the glycoprotein granulin, which promotes the production of periostin by HSCs and results in the formation of a fibrotic microenvironment that supports tumour growth.^92^

### Ageing, tumour progression and liver metastasis

Changes in the TME that occur during ageing are now being recognised to play an important role in tumour progression and can have profound effects on the development of metastases.^93^ Such changes include the reprogramming of the fibroblast secretome, which can enhance metastatic spread by mediating physical alterations in the ECM that promote enhanced migration and invasiveness^94^ Interestingly, age-related changes were also associated with a reduction in the motility of certain immune cell populations, resulting in a change in the immune environment.^94^ Although these immune changes were not directly associated with a reduction in metastatic capacity, several other studies have identified age-related changes in the immune microenvironment that could influence priming of the pre-metastatic niche and metastatic progression.^95^ The link between age-associated inflammation, priming of the metastatic niche and activation of resident fibroblasts is likely to play an important role in metastatic outgrowth. For example, in a mouse model of PDAC, the aged liver provides a permissive inflammatory environment that supports the activation of HSCs to secrete factors that promote the outgrowth of DTCs.^78^

## Therapeutic opportunities

Important new advances in our understanding of the TME have unveiled potential new therapeutic options, and a number of clinical trials that target interactions between tumour cells and stromal cells are underway in the metastatic disease setting and have previously been reviewed.^8,29^

### Targeting stromal cells

Inhibiting the recruitment of immune cells that drive a pro-tumorigenic environment as well as inhibition of their effector signalling pathways, or their re-education to anti-tumour phenotypes, have all been shown to prevent metastases in preclinical models.^8^ Similar approaches have been undertaken to target CAFs, by depleting specific subtypes or reprogramming them back to their resting state, or by targeting their activation or downstream effectors.^29^ For example, metastatic outgrowth following primary tumour resection was delayed following treatment with a vaccine that eliminates FAP^+^ CAFs.^96^ Targeting FAP and thereby reversing the CAF-induced pro-inflammatory tumour environment has been the focus of many studies, although the results from clinical studies in patients with advanced metastatic disease have been disappointing with limited evidence of efficacy. Further work on understanding the differences in the TME at the metastatic site is required to help optimise potential therapeutic benefit.

### Targeting CAF-derived factors

Within the TME, CAFs are a major source of TGF-β, which plays a key role in controlling anti-tumour immunity. In metastatic urothelial cancer, lack of response to the checkpoint inhibitor anti-programmed death-ligand 1 in clinical trials was linked to TGF-β signalling in fibroblasts and the consequent presence of CD8^+^ T-effector cells in the peritumoural stroma (rather than the tumour parenchyma).^97^ In a model of metastatic colorectal cancer, inhibition of TGF-β initiated a cytotoxic T cell response, and, importantly, in both these studies, inhibition of TGF-β signalling enhanced the activity of immune checkpoint inhibitors.^98^ Although direct effects on metastatic outgrowth have not been reported, effective targeting of other CAF-derived factors, including CXCL12, have also been shown to enhance the effectiveness of checkpoint immunotherapy in mouse models, and work on optimising this approach for the treatment of metastatic disease will be important going forward.^99^ CAFs are also able to restrict immune cell infiltration through remodelling of the ECM, although studies in the metastatic setting are more limited.^29^

### A note of caution

It is also important to consider that many agents in development that have been designed with the intention of directly targeting tumour cells might also have an effect on the activity of stromal cells. For example, the importance of focal adhesion kinase (FAK) within the tumour cell compartment in driving tumour proliferation and metastasis is well documented and has led to the development of a number of small-molecule inhibitors, which are currently under clinical evaluation.^100^ Interestingly, fibroblast-directed deletion of FAK, in a genetically engineered mouse model of breast cancer, reduced metastasis but had no effect on primary tumour growth. Mechanistically, this result was attributed to the secretion from FAK-deficient CAFs of exosomes enriched with the tumour-suppressive microRNAs miR-16 and miR-148a, which suppressed an EMT and migratory phenotype in the cancer cells.^101^ These data unveiled an additional role for FAK signalling in the tumour stromal compartment, promoting a more migratory and metastasis-competent phenotype. In addition, in a model of PDAC, inactivation of the kinase activity of FAK in fibroblasts reduced fibrosis, immunosuppressive cell populations and tumour progression.^102^ However, by contrast, work from the Hodivala-Dilke group has shown that loss of FAK from CAFs can promote breast tumour growth through alterations in tumour metabolism.^103^ The use of different promoters to drive Cre recombinase-mediated fibroblast-specific deletion of FAK might represent targeting of distinct CAF subpopulations and explain the disparate results in these studies. However, the results highlight the need to consider the impact of treatments on both stromal and tumour-derived signals, and that subtle differences in the crosstalk between specific subpopulations of stromal cells will be important in determining the response. To this end, understanding the early interplay between immune cells, HSCs/CAFs and tumour cells during metastatic colonisation is important in developing therapeutic intervention strategies. It would not be necessary to target all of these components: a single aspect of this TME could be targeted, which would destabilise the formation of early metastases. For example, by targeting CCR5 on immune cells using a Food Drug Administration-approved antagonist (maraviroc), Adwan and colleagues^104^ were able to reduce the liver colonisation of colorectal cancer cells in an animal model. The TME should be seen as a well-balanced and evolving microenvironment that is sensitive to disruption with targeted therapies.

## Conclusions

The TME clearly plays a critical role in shaping the progression of highly aggressive metastatic disease, for which current treatment options are limited. Our increased understanding of the complex interplay between different stromal compartments and how they influence tumour cell phenotypes is key to developing more effective therapies. These endeavours are being aided by new technologies, such as single-cell RNA-sequencing. This approach has identified distinct transcriptomic profiles in micrometastatic lesions from breast cancer-patient-derived xenograft models and revealed mitochondrial oxidative phosphorylation to be the main pathway upregulated in micrometastases in these models. Notably, targeting oxidative phosphorylation could reduce metastatic seeding.^105^ Single-cell analysis of CAFs has also identified specific subsets of CAFs associated with immunosuppression and immunotherapy resistance in patients with breast cancer raising the possibility of targeting specific CAF populations to enhance the effects of immunotherapies.^106^ Such analysis also highlights the growing complexity governing the metastatic process, and future studies might benefit from incorporating a systems-level approach to help uncover key hubs that are responsible for driving disease progression.^107^

A number of organs, including the liver, appear to be privileged sites for tumour metastasis. The liver has evolved to be highly regenerative and contains a number of cell lineages (epithelial and endothelial) that can exit mitotic quiescence and proliferate to repair damage or deposit scar tissue to maintain the integrity of the tissue. Given the central role the liver plays in detoxification and how it is subject to recurrent insult/injury throughout adult life, this is a necessary evolutionary adaptation. However, this regenerative capability has also allowed it to become a prime metastatic site for CTCs. While the local immune repertoire of the sinusoid can sense tumour cells and orchestrate their killing, once a cancer cell has overcome this surveillance it can enter the space of Dissé where it co-opts local HSCs and immune cells to build a metastatic microenvironment, changing the physical and signalling parameters around these early tumour cells to facilitate their survival and promote their growth. While some of the cellular and acellular players have been identified in liver, we should not overlook the fact that these components may differ between tissues. Indeed, the process of colonisation could be very different depending on the tissue of origin of the cancer cell(s), with different signals and cells required to initiate metastasis.

The TME is highly dynamic; however, most studies monitoring metastatic spread are only able to provide a snapshot of what is happening. Intravital imaging approaches that allow imaging in both the primary and metastatic sites are now providing key insights into the dynamic temporal and spatial regulation of different stromal cell populations and how they interact with each other and the tumour cells.^43,108,109^ Taken together, the complexity and heterogeneity of signalling within the stroma mean that a pan-treatment targeting individual stromal cells is unlikely to be effective, and that tumour-specific approaches might instead be required. A greater understanding of how the reciprocal communication between immune cells and CAFs sustains a pro-tumorigenic environment provides additional impetus for combining therapies that target CAFs and immune cell functions and opens up exciting opportunities for treating patients with metastatic disease, an important area of unmet clinical need.

## Data Availability

Not applicable.
